# Encephalitis, acute renal failure, and acute hepatitis triggered by a viral infection in an immunocompetent young adult: a case report

**DOI:** 10.1186/1752-1947-3-9289

**Published:** 2009-11-21

**Authors:** Mahmoud Aboelneen Khattab, Mohammed Eslam, Mohammed Emad Abd-elfattah

**Affiliations:** 1 Department of Internal Medicine, Minia medical school, Minia University, Minia, Egypt

## Abstract

**Introduction:**

Cytomegalovirus generally causes self-limited, mild and asymptomatic infections in immunocompetent patients. An aggressive course in immunocompetent healthy patients is unusual.

**Case presentation:**

We report the case of an immunocompetent 16-year-old Egyptian boy with encephalitis, acute renal failure, and acute hepatitis triggered by viral infection with a complete recovery following antiviral treatment.

**Conclusion:**

We believe that this case adds to the understanding of the molecular biology, clinical presentation and increasing index of suspicion of many viral infections.

## Introduction

Cytomegalovirus (CMV) (from the Greek cyto-, 'cell', and -megalo-, 'large') is a viral genus of the Herpes virus group. In humans, it is commonly known as human cytomegalovirus (HCMV) or human herpes virus 5 (HHV-5) [1]. CMV belongs to the Betaherpesvirinae subfamily of *Herpesviridae*, which also includes Roseolo virus. Other herpes viruses fall into the subfamilies of Alphaherpesvirinae, including the herpes simplex virus (HSV)-1 and HSV-2 and varicella, or Gammaherpesvirinae, including Epstein-Barr virus (EBV) [1]. All herpes viruses share a characteristic ability to remain latent within the body over long periods.

HCMV infection is more common in developing countries and in communities with lower socioeconomic status and represents the most significant viral cause of birth defects in industrialized countries.

Most healthy people who are infected by HCMV after birth have no symptoms [1]. Cytomegalovirus generally causes self-limited, mild and asymptomatic infections in immunocompetent patients. Some of them develop an infectious mononucleosis or glandular fever-like syndrome [2], with a prolonged fever, and mild hepatitis. A sore throat is common. After infection, the virus remains latent in the body for the rest of the person's life. Overt disease rarely occurs unless immunity is suppressed either by drugs, infection or old-age. Initial HCMV infection, which is often asymptomatic is followed by a prolonged, unapparent infection during which the virus resides in cells without causing detectable damage or clinical illness.

Of all of the human herpes viruses described to date, CMV arguably causes the most morbidity and mortality. Until the past two decades, CMV encephalitis was considered to be a rare condition. However, since the first reports of autopsy results were published at the beginning of the acquired immunodeficiency syndrome (AIDS) epidemic [3-5], CMV encephalitis has been common in dying patients infected with human immunodeficiency virus (HIV), and remained limited to groups of immunosuppressed patients.

## Case presentation

A 16-year-old Egyptian boy was referred to our department in 2008. He was comatose with serial fits, and had an acute onset of renal failure for which one session of dialysis was done. His relatives reported an acute onset of disturbed conscious level with serial fits and low grade fever four days earlier. His past medical and drug history were unremarkable.

At presentation, he was febrile (temperature 38.0°C) and hypertensive (blood pressure 170/100 mmHg). Central venous pressure (CVP) was 13 mmHg and urine output (UOP) was 100 cc. Otherwise, in general, his neurological examination was normal except for serial fits.

Initial laboratory tests included a white blood cell count of 13.7 (4.0-11.0 × 10^3^/μL) with predominant lymphocytosis, hemoglobin 15.5 (13.5-17.5 g/dL), platelets 200 (150-400 × 10^3^/μL), aspartate aminotransferase (AST) 1003 (20-57 IU/L), alanine aminotransferase (ALT) 1200 (21-72 IU/L), total bilirubin 1.1 (0.0-1.5 mg/dL), direct bilirubin 0.6 (0.0-0.8 mg/dL), alkaline phosphatase 120 (30-136 IU/L), international normalized ratio (INR) 1 and prothrombin time 11 (10.0-13.5 s), serum albumin 4 (3.5-6 mg/dL), random blood sugar 250 (140-199 mg/dL), creatinine 10 (0.6-1.2 mg/dL), urea 330 (15-45 mg/dL), Na^+ ^136 (135-145 mmol/L), K^+ ^3.6 (3.5-5), and calcium 9 (9-11 mg/dL). For acute hepatitis A IgM, hepatitis B surface antigen and anti-HB core antibody, and HCV-antibodies by third generation enzyme linked immunosorbent assay (ELISA), hepatitis C RNA by polymerase chain reaction (PCR) as well as HIV-antibodies, serologies were negative. Also, an autoimmune screen, anti-double stranded DNA, pANCA and cANCA, anti-nuclear antibody, and anti-smooth muscle antibody, were all negative. A surface echocardiogram revealed an ejection fraction of 75-80.

Serum, liver and renal biochemistry was normal 4 days earlier (AST 32 IU/L, ALT 40 IU/L, total bilirubin 0.5 mg/dL) when the patient was admitted to the fever hospital. He was receiving ceftriaxone 2 g intravenously every 24 hours, phenytoin 100 mg intravenously every 8 hours, and paracetamol 0.5 g every 6 hours.

**Figure 1:**
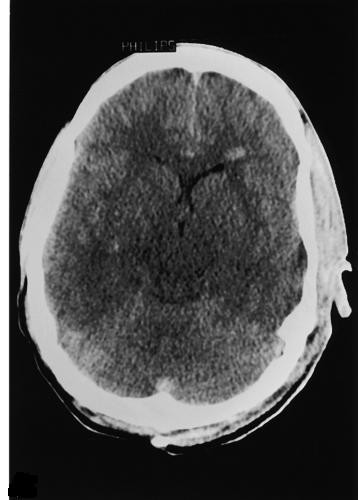
**A computed tomography scan at admission showing mild brain edema**.

**Figure 2:**
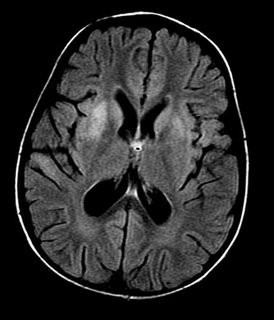
**Magnetic resonance imaging at the time of admission showing increased signal intensity on T2 weighted images with symmetrical bilateral putaminal vasogenic edema with mild restriction on diffusion and no bleeding**.

**Figure 3:**
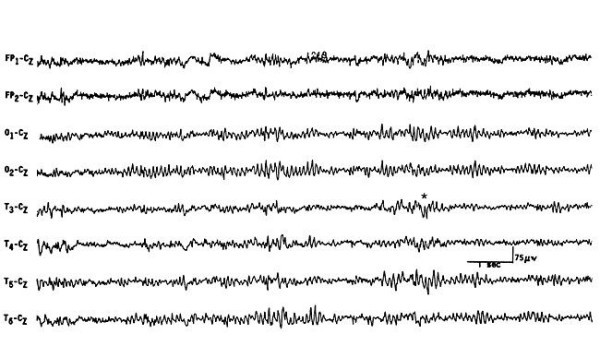
**Electroencephalography of the patient at the time of admission showing slowing background**.

An abdominopelvic ultrasound was unremarkable, and chest X-ray was normal. Brain computed tomography (CT) scan without contrast revealed mild brain edema (Figure 1), magnetic resonance imaging revealed an encephalitic pattern (Figure 2), and electroencephalography (EEG) revealed an encephalitic pattern (Figure 3). Cerebrospinal fluid examination returned the following results: glucose 160 mg/dL, protein 20 (20-40 mg/dL), chlorides 717 (720-750 mg/dL), and cells 70/mm^3^, which were mainly lymphocytes.

Serology tests were negative for influenza A and B, respiratory syncytial virus (RSV), Mycoplasma pneumoniae, chlamydia, Coxiella burnetii, adenovirus, enterovirus, and antistreptolysin O titer (ASOT). A serology test for EBV by PCR was also negative. A serology test for CMV confirmed acute infection, with a 10-fold rise in CMV-specific IgM. Polymerase chain reaction (PCR) for (CMV) was also positive in the serum and the CSF.

Because of the impaired renal function, the patient was started on acyclovir 10 mg/day. Lamotrigine was chosen as antiepileptic drug as it is safe to use in patients with elevated liver enzymes, in addition to other supportive measures such as glycerol as a cerebral dehydrating measure, and antibiotics.

**Figure 4:**
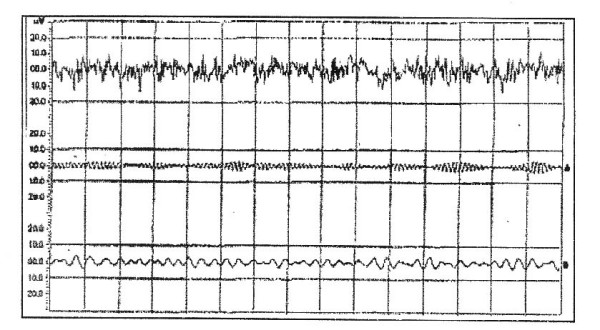
**Electroencephalography of the patient at the time of discharge: normal finding**.

Over the next three days, fits were controlled. On the third day, the patient started to regain consciousness and became fully conscious by day 11. Renal function returned to normal (without dialysis) after 17 days. Liver enzymes returned to normal after 20 days. A follow-up EEG revealed no abnormality (Figure 4). The patient left hospital and made a complete recovery with no residual effects (Table 1).

**Table 1:** Characteristics of the patient at the time of admission and discharge

	At the time of admission	At the time of discharge
Blood pressure (systolic/diastolic)	170/110	120/80
AST (IU/L)	1003	35
ALT (IU/L)	1200	40
Creatinine (mg/dL)	10	0.5
Urea (mg/dL)	330	30
Random blood sugar (mg/dL)	250	100

## Discussion

CMV generally causes self-limited, mild and asymptomatic infections in immunocompetent patients. In these patients, CMV infections are characterized as mononucleosis-like syndrome with fever, cervical adenopathy and elevation of liver enzymes [6]. It may be organ-specific or affect multiple organs.

There are limited data on the molecular mechanisms responsible for the pathogenesis of tissue damage caused by CMV. Recent investigations into the molecular biology of CMV have revealed the presence of many viral gene products, which appear to modulate host inflammatory and immune responses [7]. Several CMV genes interfere with normal antigen processing and generation of cell-mediated immune responses, and these may be related to changes in the nature of the virus related disease as it seems to take an aggressive course in immunocompetent healthy patients.

## Conclusion

We describe a rare case of encephalitis, acute renal failure, and acute hepatitis triggered by a CMV infection in an immunocompetent young adult. This case adds to the understanding of the molecular biology, clinical presentation and increasing index of suspicion of many viral infections especially CMV in patients presenting with effects in multiple organs.

## Abbreviations

AIDS: acquired immunodeficiency syndrome; ALT: alanine aminotransferase; AST: aspartate aminotransferase; CT: computed tomography; ELISA: enzyme linked immunosorbent assay; INR: international normalized ratio; Na: sodium; K: potassium; TB: tuberculosis; MRI: magnetic resonance imaging; EEG: electroencephalography; PCR: polymerase chain reaction; CSF: cerebrospinal fluid; CMV: cytomegalovirus; EBV: Epstein-Barr virus; RSV: respiratory syncytial virus; ASOT: antistreptolysin O titer; CVP: central venous pressure; HSV 1, 2: Herpes simplex virus 1, 2; HCMV: human cytomegalovirus; c-ANCA: cytoplasmic antineutrophil cytoplasmic antibodies; p-ANCA: perinuclear antineutrophil cytoplasmic antibodies; UOP: urine output.

## Competing interests

The authors declare that they have no competing interests.

## Authors' contributions

MAK was involved in study concept and design, patient care, literature review, data analysis, drafting and revising the manuscript. ME and MED were involved in study concept and design, patient care, literature review, and drafting the manuscript. All authors have read and approved the final version of the manuscript.

## Consent

Written informed consent was obtained from the patient for publication of this case report and any accompanying images. A copy of the written consent is available for review by the Editor-in-Chief of this journal.

## References

[B1] Ryan KJ Ray CG (Eds) Sherris Medical Microbiology 2004 4 McGraw Hill 556 566-569

[B2] Bottieau E Clerinx J Enden E Van den Van Esbroeck M Colebunders R Van Gompel A Ende J Van den Infectious mononucleosis-like syndromes in febrile travelers returning from the tropics J Travel Med 2006 13 4 191 197 10.1111/j.1708-8305.2006.00049.x 16884400

[B3] Hui AN Koss MN Meyer PR Necropsy findings in acquired immunodeficiency syndrome: a comparison of premortem diagnoses with postmortem findings Hum Pathol 1984 15 670 676 10.1016/S0046-8177(84)80293-2 6086491

[B4] Snider WD Simpson DM Nielsen S Gold JW Metroka CE Posner JB Neurological complications of acquired immune deficiency syndrome: analysis of 50 patients Ann Neurol 1983 14 403 418 10.1002/ana.410140404 6314874

[B5] Welch K Finkbeiner W Alpers CE Blumenfeld W Davis RL Smuckler EA Beckstead JH Autopsy findings in the acquired immune deficiency syndrome JAMA 1984 252 1152 1159 10.1001/jama.252.9.1152 6471338

[B6] Hadaya K Kaiser L Rubbia-Brandt L Gervaix A Diana A Ganciclovir for severe cytomegalovirus primary infection in an immunocompetent child Eur J Clin Microbiol Infect Dis 2004 23 218 220 10.1007/s10096-003-1079-z 14767679

[B7] Schleiss MR McVoy MA Overview of congenitally and perinatally acquired cytomegalovirus infections: recent advances in antiviral therapy Expert Rev Anti Infect Ther 2004 2 3 389 403 10.1586/14787210.2.3.389 15482204

